# MixSeg: a lightweight and accurate mix structure network for semantic segmentation of apple leaf disease in complex environments

**DOI:** 10.3389/fpls.2023.1233241

**Published:** 2023-09-13

**Authors:** Bibo Lu, Jiangwen Lu, Xinchao Xu, Yuxin Jin

**Affiliations:** School of Computer Science and Technology, Henan Polytechnic University, Jiaozuo, China

**Keywords:** MixSeg, CNN, Transformer, MLP, fruit leaf disease, semantic segmentation

## Abstract

**Introduction:**

Semantic segmentation is effective in dealing with complex environments. However, the most popular semantic segmentation methods are usually based on a single structure, they are inefficient and inaccurate. In this work, we propose a mix structure network called MixSeg, which fully combines the advantages of convolutional neural network, Transformer, and multi-layer perception architectures.

**Methods:**

Specifically, MixSeg is an end-to-end semantic segmentation network, consisting of an encoder and a decoder. In the encoder, the Mix Transformer is designed to model globally and inject local bias into the model with less computational cost. The position indexer is developed to dynamically index absolute position information on the feature map. The local optimization module is designed to optimize the segmentation effect of the model on local edges and details. In the decoder, shallow and deep features are fused to output accurate segmentation results.

**Results:**

Taking the apple leaf disease segmentation task in the real scene as an example, the segmentation effect of the MixSeg is verified. The experimental results show that MixSeg has the best segmentation effect and the lowest parameters and floating point operations compared with the mainstream semantic segmentation methods on small datasets. On apple alternaria blotch and apple grey spot leaf image datasets, the most lightweight MixSeg-T achieves 98.22%, 98.09% intersection over union for leaf segmentation and 87.40%, 86.20% intersection over union for disease segmentation.

**Discussion:**

Thus, the performance of MixSeg demonstrates that it can provide a more efficient and stable method for accurate segmentation of leaves and diseases in complex environments.

## 1 Introduction

Apples are rich in nutritional value and are one of the most important cash crops in the world. However, apple leaves are often affected by pests and diseases, and failure to identify and prevent diseases promptly can easily lead to a decrease in fruit quality and yield, causing severe economic losses to growers (Bansal et al., 2021; Yogeshwari and Thailambal, 2021; Aggarwal et al., 2023). In reality, the identification of crop diseases usually relies on manual work (Chen et al., 2022; Mu et al., 2022). The manual approach has many drawbacks, such as excessive subjectivity, low efficiency, and high recognition error rate (Barman et al., 2020; Xu et al., 2023). Therefore, computer vision technology can be used to identify diseases quickly and accurately, which plays a crucial role in effective disease prevention and higher apple fruit quality and yield.

In recent years, with the rapid development of computer vision technology, deep learning had gained much attention in crop disease identification. More and more researchers used semantic segmentation methods for crop disease recognition. For example, (Storey et al., 2022) used ResNet-50 and MobileNetV3 as the backbone of Mask-RCNN and then segmented apple disease leaf disease images separately to test their suitability for this task. (Divyanth et al., 2023) researched the advantages and disadvantages of the semantic segmentation networks SegNet, U-Net and DeepLabv3+, and chose U-Net and DeepLabv3+ for segmentation of corn leaves and disease spots, respectively. (Wu et al., 2021) tested the effect of DeepLabv3+ with different backbone networks, Xception-65, Xception-71, ResNet-50, and ResNet-101, on the performance of leaf and disease spot segmentation of hydroponic lettuce, providing a guide for an automatic selection and segmentation device for hydroponic lettuce. (Agarwal et al., 2021) chose ResNet as the encoder of U-Net to achieve effective segmentation of crop disease leaf images. (Yuan et al., 2022) replaced the backbone network of DeepLabv3+ with the lightweight MobileNetV2 to reduce the training time of the model and was able to segment the leaf veins in real-time. Therefore, semantic segmentation methods are suitable for recognizing crop leaves and diseases in complex environments.

Currently, the mainstream semantic segmentation models are mainly based on convolutional neural network (CNN) structure and Transformer structure. CNN achieved a certain degree of offset, scale, and distortion invariance by forcing the capture of local priors using local perceptual fields. For the same target appearing at different locations in the image, all feature representations with some similarity can be extracted by CNN (Zeiler and Fergus, 2014; He et al., 2015). In addition, CNN had a hierarchical learning model, from simple low-level textures to higher-order semantic patterns. This property of CNN allowed for strong robustness and generalization when dealing with problems such as target recognition (Simonyan and Zisserman, 2014). Therefore, CNN was useful for extracting and optimizing local leaf and disease features in apple leaf disease image segmentation. However, the convolution operation focuses only on local regions, which can lead to general limitations of CNN for modeling relationships between distant pixels (Kuo, 2016; Hu et al., 2018). (Simonyan and Zisserman, 2014) extended the ability to extract global features by stacking deeper networks to increase the field of perception. While this alleviated the general limitation of CNN for modeling direct long-range relations, too deep layers introduced the problem of gradient degradation, which led to degradation of the model’s performance (He et al., 2016). Some researchers had started using self-attention instead of convolution to extract global features (Hu et al., 2019; Ramachandran et al., 2019).

Transformer (Vaswani et al., 2017) was first applied in NLP and gradually received attention in computer vision due to its excellent performance in processing long text sequences. Unlike CNN, Transformer used self-attention to learn the relationship between pixels and regions. It can perform spatial transformations and extract feature dependencies between distant pixels, so Transformer had more flexibility. (Dosovitskiy et al., 2020) first proposed the visual Transformer ViT, which treated an image as a set of sequences and uses self-attention for global modeling. Subsequently, a series of Transformer-based semantic segmentation models started to appear, such as SegFormer (Xie et al., 2021a), PoolFormer (Yu et al., 2022), and SETR (Zheng et al., 2021). However, the semantic segmentation networks with self-attention as the global modeling paradigm had some problems. For example, self-attention had a quadratic computational complexity for the input token sequence, and therefore, the network was not conducive to high-resolution input. In addition, self-attention had many parameters and was prone to overfitting when the dataset is small (Huang et al., 2019; Wang et al., 2020; Guo et al., 2022). Moreover, self-attention cannot encode the position and cannot recover the target’s position information in the decoder. These problems eventually led to less efficient network segmentation and poor segmentation results. Multi-layer perception (MLP) was a typical feedforward neural network consisting of multiple layers of neurons, each fully connected to the previous layer. Compared to self-attention, MLP aimed to establish weights for all features. Thus it possessed a more robust ability than self-attention to extract the dependencies of features that show long-range (Ding et al., 2021). Moreover, MLP can be viewed as a combination of multiple linear transformations and nonlinear functions, and it only needed to learn the weights and biases of each layer, so MLP was less susceptible to noise and erroneous inputs and was more stable in computation. However, the traditional MLP had many parameters, was computationally complex, and was also prone to overfitting. In summary, the correct use of local prior, global dependence and control of computational complexity are the keys to improving the performance of semantic segmentation models.

In response to the above problems of single structured networks, the goal of this study was to design a mix structure semantic segmentation model by extracting the core strengths of CNN, Transformer and MLP architectures to improve the global modeling efficiency of the model while maintaining a strong mastery of detailed features and being more lightweight. Specifically, MixSeg consisted of three key components: Mix Transformer, position indexer (PI), and local optimization module (LOM). Mix Transformer aimed to leverage the local feature injection of CNN, the stability of global modeling structure of Transformer, and the ability of MLP to establish complete global dependency at a smaller computational cost for extracting more comprehensive global features. The role of the position indexer was to accurately preserve the position information of target leaves during the downsampling process in order to enhance the model’s anti-interference capability and segmentation performance. LOM, on the other hand, focused on optimizing the extraction of local features such as disease leaf edges and details. In summary, MixSeg was a mix structure model applied to segment apple leaves and diseases in complex environments. Compared with the single structure model, it was capable of better segmenting target leaves and diseases while being more lightweight and suitable for practical applications. The main contributions were as follows:

(1) A lightweight and accurate mix structure semantic segmentation model (MixSeg) was designed to improve the segmentation performance of apple leaves and disease spots in complex environments with fewer parameters and computational effort.
(2) A novel Mix Transformer was designed, which uses the depthwise separable convolution of the residual for local bias injection, and the designed MMLP to reduce the amount of computation and establish global dependencies.
(3) The PI was designed to dynamically index absolute position information on the feature map and can be independent of variable length inputs.
(4) The LOM was designed to enhance the model’s ability to represent local features, optimize the segmentation effect of leaf edges and extract more tiny spots.

The rest of the paper was organized as follows: the dataset and the details of the proposed MixSeg were presented in Section 2. Then, the experimental results were presented and analyzed in Section 3. Next, the limitations of MixSeg were analyzed by discussing the proposed method in Section 4. Finally, the work of this study was summarized in Section 5 and future research directions are envisioned.

## 2 Materials and methods

### 2.1 Data collection and annotation

The data for this work were obtained from Northwest Agriculture and Forestry University manual photpgraphy was adopted to obtain images of apple leaf diseases in real outdoor scenarios. A total of two common apple leaf diseases were collected, namely apple alternaria blotch and apple grey spot. In addition, the weather in which these dataset images were taken includes both sunny and rainy days, which enhanced the dataset’s diversity and authenticity.

**Figure 1:**
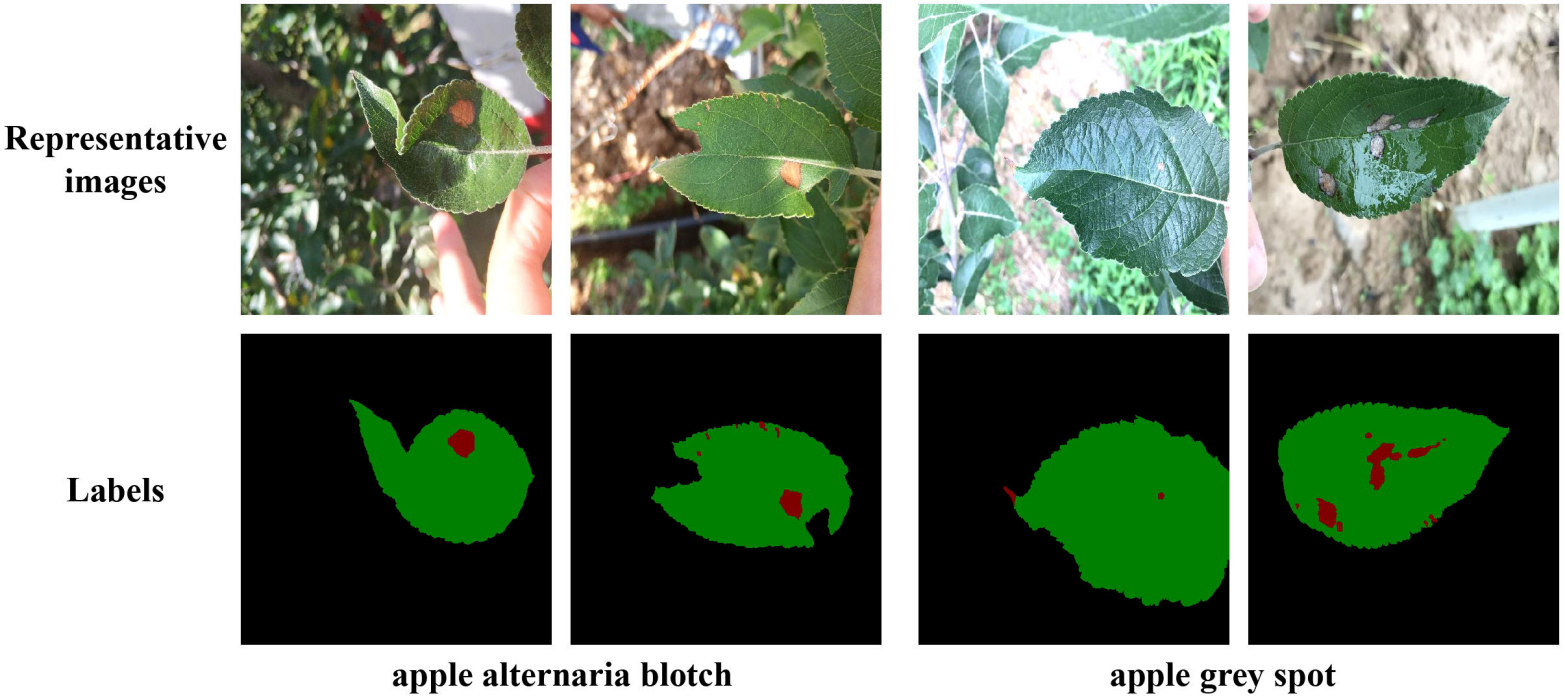
Representative images of two types of apple leaf disease spots and their corresponding labels. In the labels, the black, green and red parts represent the background, the leaf and the diseased area, respectively.

To improve the training accuracy, we used the professional semantic segmentation labeling software Labelme to label the original images under the guidance of experts. We finely labelled the edges of each leaf and disease by a pixel-by-pixel approach. Then corresponding label files was generated, where the background, leaf, and disease pixel values were set as 0, 1, 2, respectively. The partial images and labels of the two apple leaf datasets were illustrated in **Figure 1**, where the black, green, and red parts of the label represented the background, leaf and disease area, respectively.

### 2.2 Data augmentation

**Figure 2:**
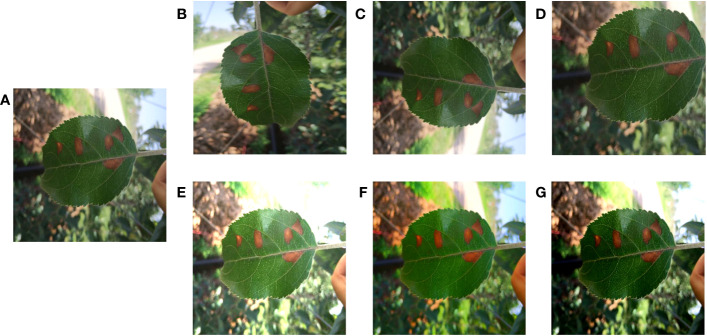
Image enhancement. **(A)** Original image. **(B)** Image rotation. **(C)** Image flip. **(D)** Image crop. **(E)** Saturation enhancement. **(F)** Brightness enhancement. **(G)** Contrast enhancement.

For deep learning methods, too little data volume tended to lead to model overfitting and poor generalization. (Lee et al., 2019). Therefore, it was necessary to use data augmentation methods to properly extend the two apple leaf datasets. A total of six data enhancement methods were applied: (1) Geometric deformation: the original image and labels were cropped, randomly folded, or rotated simultaneously to obtain new images and labels. (2) Chromaticity change: the original image brightness, contrast, or saturation was changed to obtain a new image, and the labels remain unchanged. These data enhancement methods simulate the changes in shooting angle and illumination during data acquisition, which can improve the model’s robustness and generalization ability. The apple alternaria blotch disease dataset had 256 original images and 1536 enhanced images, totaling 1792 images. Apple grey spot disease dataset had 162 original images and 972 enhanced images, totaling 1134 images. Taking the apple alternaria blotch leaf image dataset as an example, the enhanced images were shown in **Figure 2**.

### 2.3 Design for MixSeg

**Figure 3:**
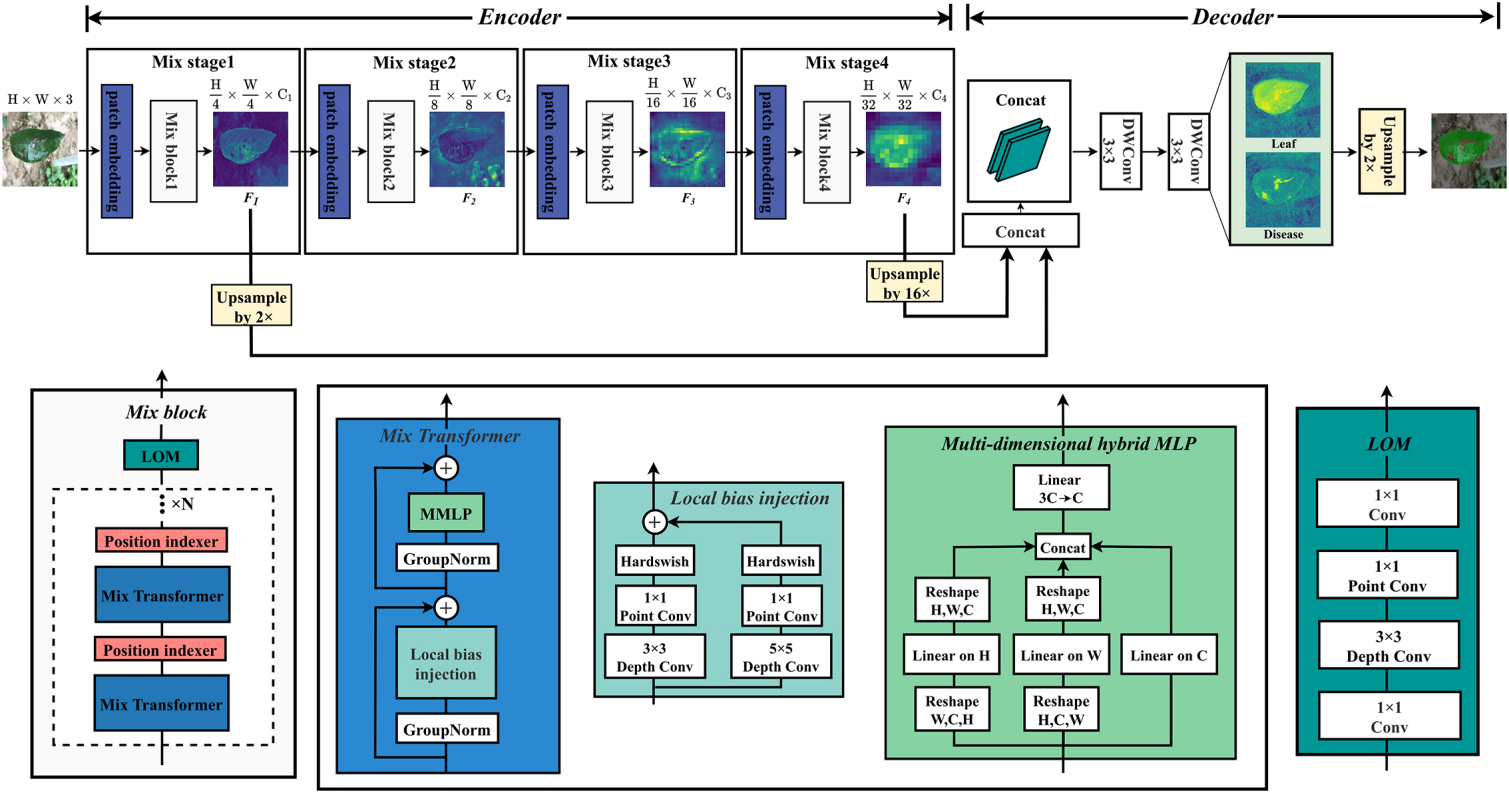
MixSeg overall architecture. The encoder of MixSeg consists of four MixStages, each with a patch embedding for feature serialization and a Mix block for feature extraction. Mix transformer, PI, and LOM together form the Mix block. In addition, N represents the number of stacks of Mix Transformer and PI, which is determined by the size of the network designed in this paper. In the decoder, DWConv represents the depthwise separable convolution. The decoder fuses Mix Stage1 and Mix Stage4 output results and outputs the fine-grained segmentation results by two DWConv.

In order to improve the segmentation accuracy and efficiency, a mix structure network MixSeg combining the advantages of CNN, Transformer and MLP was designed, the overall structure was shown in **Figure 3**. MixSeg adopted an encoding-decoding structure. In the encoder, the network consisted of four sets of Mix stages. In each Mix stage, it consisted of a patch embedding and Mix block. In addition, the core components of Mix block consisted of Mix Transformer, PI, and LOM designed by us. In the decoder, shallow and deep features were fused and accurate segmentation results were output by two 3×3 depthwise separable convolutions.

Specifically, the image size of the input network was 512×512×3. We used a 7×7 patch in Mix stage 1, and stride was set to 4. The token serialization of the input image was performed in an overlapping manner to reduce the parameters of the initial input network. Each token was reshaped into a 147 dimensional vector and mapped to a *C*_1_ dimensional embedding through a linear layer. Immediately afterward, the extracted token sequence sets were fed into Mix Transformer for global modeling. In particular, a PI was inserted after each Mix Transformer to prevent the absolute position information of the target from being lost when building global dependencies. Then, the output of the Mix Transformer was optimized by a LOM for local features. After the Mix stage 1, the feature map *F*_1_ with dimension $\left(\frac{H}{4}\times \frac{W}{4}\times C_{1}\right)$ was output, and the inter-level quadruple downsampling was completed. In addition, the channel dimensions *C_i_* (i=1, 2, 3, 4) of the output feature maps in MixSeg-T and MixSeg-S were [32, 64, 160, 256], and MixSeg-M, MixSeg-L were [64, 128, 256, 512]. Other Mix stages all use 3×3 size patches with stride 2, the inter-level double downsampling was performed, and the multi-scale feature maps *F*_2_, *F*_3_, *F*_4_ with $\left\{\frac{1}{8},\frac{1}{16},\frac{1}{32}\right\}$ of the original input are output. *F*_1_ and *F*_4_ perform feature fusion after 2x and 16x upsampling, and finally produced fine segmentation results by two depthwise separable convolutions. The network innovations were explained in detail in the remainder of this section. Mix Transformer, PI, and LOM were introduced in Section 2.3.1, 2.3.2, and 2.3.3, respectively.

#### 2.3.1 Mix Transformer

Traditional Transformer architecture networks, such as ViT, usually consisted of self-attention and channel MLP to achieve spatial and channel information mixing. When pre-trained on a large dataset, these traditional networks performed well on image recognition tasks. However, in real production situations, data acquisition and labeling were difficult and suffer from insufficient sample size. In addition, in segmentation tasks, the image size of the input network was usually large, and self-attention for global modeling requires dealing with many long-range pixel dependencies. Therefore, semantic segmentation networks used self-attention as the design paradigm were usually more complex, and they were prone to overfitting phenomena on small-scale datasets, which was not conducive to applications in real production. In this subsection, we proposed a new Transformer design paradigm named Mix Transformer. It utilized depthwise separable convolution to inject local bias. It further utilized the designed multi-dimensional hybrid MLP (MMLP) to reduce the computational complexity and built a complete global dependency in multiple dimensions to make the model better match small datasets. The structure of the Mix Transformer was demonstrated in **Figure 3**.

As shown in **Figure 3**, the Mix Transformer maintained the basic architecture of the Transformer, which was composed of two main residual blocks. In particular, local bias injection was performed in the first main residual block, which consisted of GroupNorm and 3×3, 5×5 depthwise separable convolution of residuals connected. Global feature dependencies were established in the second main residual block, which consisted of GroupNorm and MMLP.

Local biasing was a common technique in CNN, which summed a fixed bias value to each position of the input data convolved by an additive operation (Zhuang et al., 2019). Such an operation allowed each convolutional kernel to learn different features and produced corresponding responses to various input data locations. Self-attention did not have local bias, which made some noise and interference in the input data may affect the computational results. Therefore, we chose depthwise separable 3×3 and 5×5 residual convolutions instead of self-attention to inject local bias to the token sequence set. Specifically, the first main residual block can be expressed as:


(1) $$Y_{i}=\left(DWConv_{3\times 3}\left(Norm\left(X_{i}\right)\right)+DWConv_{5\times 5}\left(Norm\left(X_{i}\right)\right)\right)+X_{i}$$

where *X_i_* (i=1, 2, 3, 4) was the output of the patch embedding in each Mix stage, *X_i_* ∈ *R* ^(^ *^N_i_^* ^×^ *^C_i_^* ^)^. *N* represented the number of token sequences, *C* stood for their sequence dimension. Then, *X_i_* was reshaped as *X_i_* ∈ *R* ^(^ *^H_i_^* ^×^ *^W_i_^* ^×^ *^C_i_^* ^)^. Thus after the input local bias injection *Y_i_* ∈ *R* ^(^ *^H_i_^* ^×^ *^W_i_^* ^×^ *^C_i_^* ^)^. In addition, DWConv presented the depthwise separable convolution. 3×3 and 5×5 DWConv required few parameters to inject rich multiscale local bias to the set of token sequences extracted by patch embedding.

**Figure 4:**
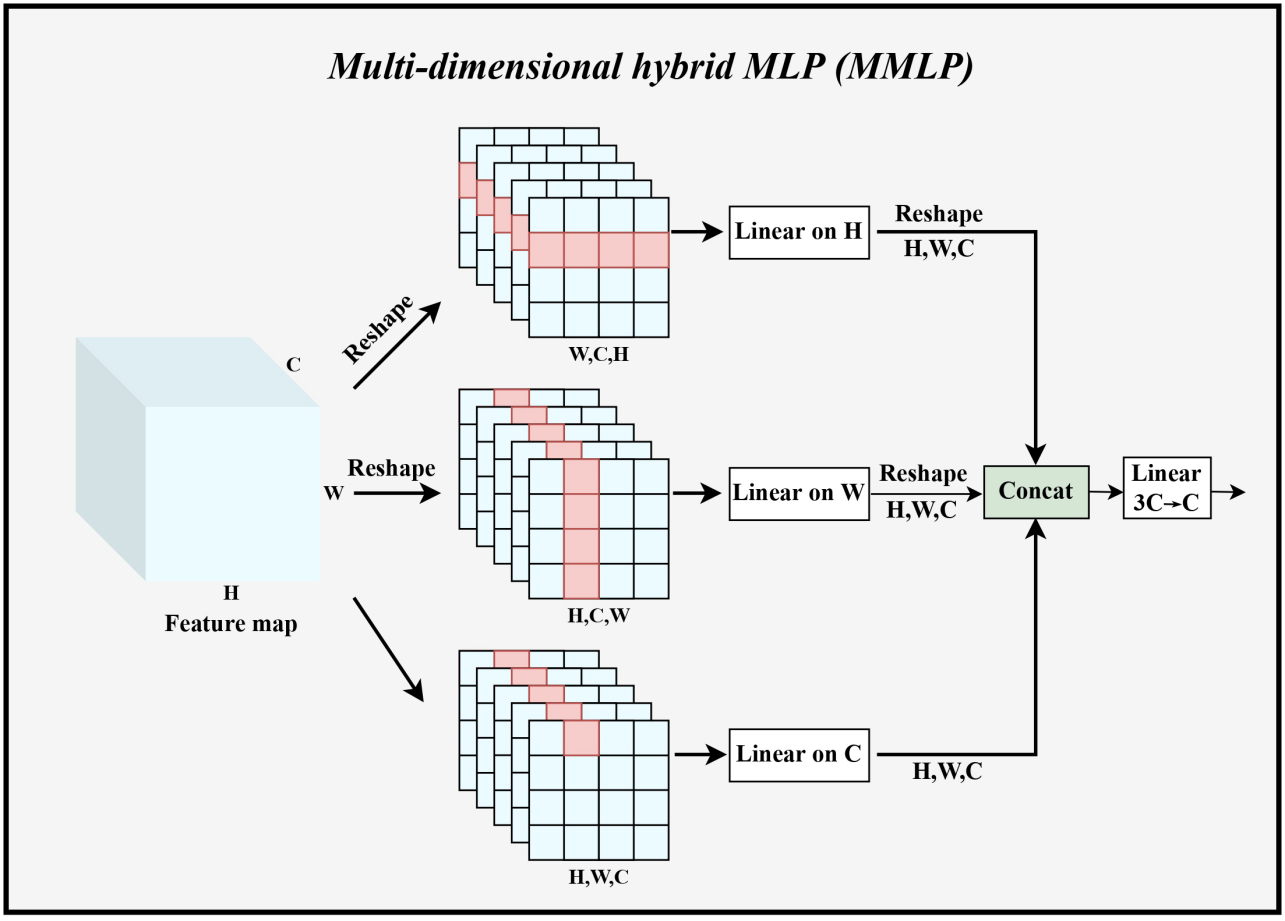
The specific implementation process of MMLP. Linear represents linear feature mapping, Concat indicates stitching the fused feature maps in the channel dimension, and Reshape denotes changing the shape of the feature maps to the specified dimension.

Transformer architecture usually used channel MLP for mixing channel information. Channel MLP interacted with all the elements in each token, so the computational complexity was large when the token sequence was long in dimension, and overfitting was likely to occur. In addition, channel MLP can only mix information on the channels. Therefore, in the second main residual block, we designed MMLP to improve the original MLP by reducing the computational effort and jointly establishing global dependencies in multiple dimensions to make the model less prone to overfitting when training on small datasets. **Figure 4** illustrated the specific architecture of MMLP.

Specifically, *Y_i_* obtained from Equation 1 was used as input. Taking the branching path in the *H* dimension as an example, this data was reshaped as *Y_H_i* ∈ *R* ^(^ *^W_i_^* ^×^ *^C_i_^* ^×^ *^H_i_^* ^)^, and then linear feature mapping in the *H* dimension was performed to output $\hat{Y_{H^{i}}}$ , where the *H* dimension shared the weight *W_W_i* ∈ *R* ^(^ *^H_i_^* ^×^ *^H_i_^* ^)^. The same calculation was used in the *W*, *C* dimension. Immediately afterward, the outputs of these three branches were fused in the channel dimension. Then the number of channels was adjusted from 3*C_i_* to *C_i_*. The computation of the second component can be expressed as:


(2) $$\begin{array}{c} Y_{H^{i}}=Reshape\left(W_{i},C_{i},H_{i}\right)\left(Y_{i}\right),\forall i\\ Y_{W^{i}}=Reshape\left(H_{i},C_{i},W_{i}\right)\left(Y_{i}\right),\forall i \end{array}$$

(3) $$\begin{array}{c} \hat{Y_{H^{i}}}=Reshape\left(H_{i},W_{i},C_{i}\right)Linear\left(H_{i},H_{i}\right)(Y_{H^{i}}),\forall i\\ \hat{Y_{W^{i}}}=Reshape\left(H_{i},W_{i},C_{i}\right)Linear\left(W_{i},W_{i}\right)(Y_{W^{i}}),\forall i\\ Y_{C^{i}}=Linear\left(C_{i},C_{i}\right)\left(Y_{i}\right),\forall i \end{array}$$

(4) $$Z_{i}=Linear\left(3C_{i},C_{i}\right)\left(Concat\left(\hat{Y_{H^{i}}},\hat{Y_{W^{i}}},Y_{C^{i}}\right)\right),\forall i$$

Such a design of MMLP can significantly reduce the number of parameters and computation, and can establish global dependencies in different dimensions at the same time, and then summarize this global information in the mixing stage to achieve a more complete global dependency establishment. The number of parameters of MMLP module can be calculated as:


(5) $$Params_{MMLP}=H^{2}+W^{2}+C^{2}+3C^{2}$$

where the parameter of 3*C* ^2^ was used in the mixing phase of multi-dimensional information. In contrast, the parameter number of conventional MLP module was: 


(6) $$Params_{MLP}=2\tau {\left(HW\right)}^{2}$$

where 2 represented the two fully connected processes of expansion to compression, *τ* being the ratio of the expansion of the MLP layer. In addition, the computational complexity of MMLP module can be expressed as:


(7) $$FLOPs_{MMLP}=HWC\left(H+W+C\right)+3HWC^{2}$$

the computational complexity of MLP module can be expressed as:


(8) $$FLOPs_{MLP}=2\tau {\left(HW\right)}^{2}C$$

among them, the product of *H* and *W* represents the number of tokens, which was denoted as *N*. It can be found that the computational complexity of an MMLP grows with $N\sqrt{N}$ , and the MLP increases with *N* ^2^.

In summary, Mix Transformer was designed to input local bias to the network and model it globally by building a depthwise separable convolution of residuals and MMLP. Compared with the traditional Transformer architecture, Mix Transformer had lower computational complexity, which made the model lighter and, thus, more suitable for small datasets.

#### 2.3.2 Position indexer

**Figure 5:**
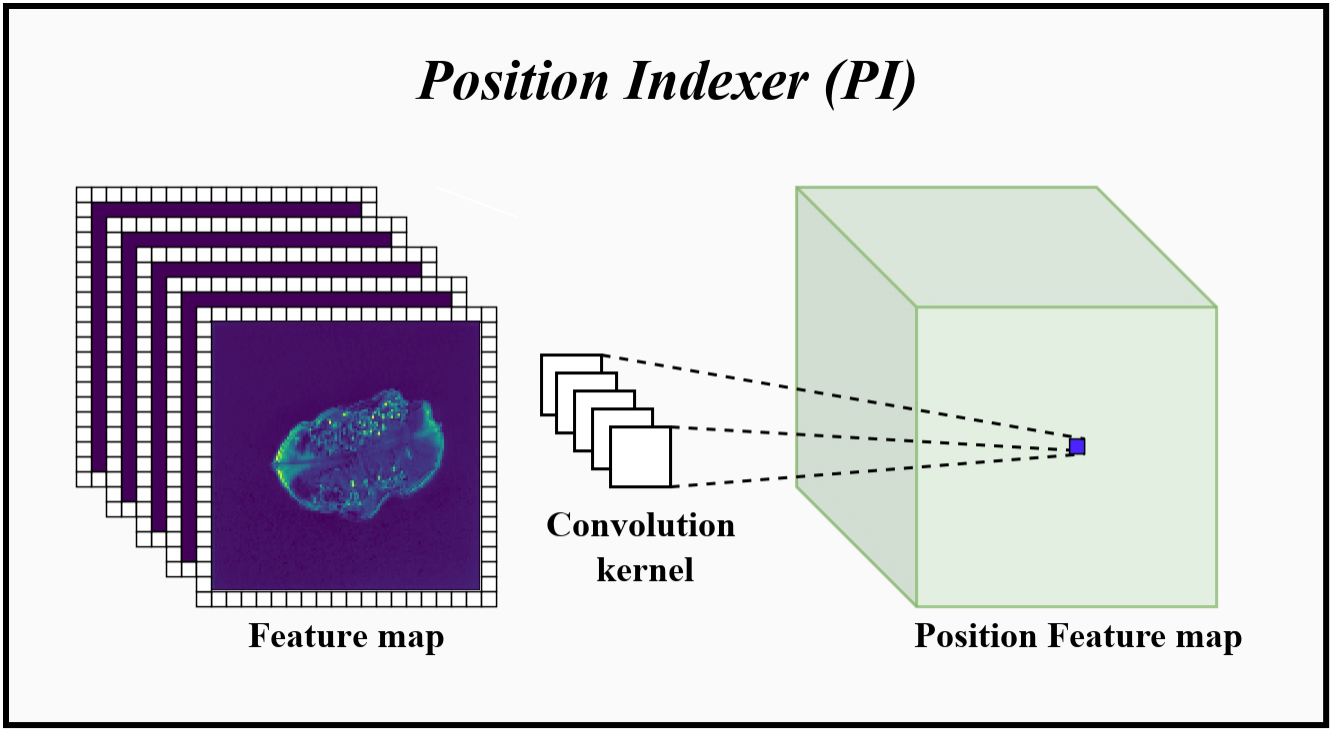
The specific implementation process of PI. Convolution kernel represents the convolution kernel group for position information indexing. Before entering the PI, the feature map will add a set of padding for learning position information around it. Then, use these convolution kernels to perform 2D group convolution on the feature map, index the position information in each channel dimension and perform fusion.

Traditional Transformer architectures implemented location information awareness by adding absolute location codes to each input token (Dosovitskiy et al., 2020). These location encodings were learnable and efficient but were disrupted when the Transformer processes longer inputs, made it impossible to implement a pyramidal downsampling pattern while preserving absolute location information. For example, ViT used a downsampling rate of 16 times at each stage of the model to maintain the order of these position-encoded vectors, which made it unable to handle multi-scale target features. Therefore, the PI that can dynamically change with the input size was proposed in this subsection, which enabled the encoder to perform multi-scale downsampling while retaining absolute position information. The schematic diagram of the PI was displayed in **Figure 5**.

In CNN, when adding different amounts of zero-padding to the input data, the convolution can predict the absolute position information of the input by learning the position and amount of zero-padding (Islam et al., 2020). Therefore, as shown in **Figure 5**, the PI performed dynamic position indexing with the padding around the input token sequence group as the domain condition. Specifically, taking *Z_i _* of Equation 4 as the input, we first added a set of padding around *Z_i _* for learning the position information, and the dimension of this padding was equal to $\frac{k-1}{2}$ , and *k* was the convolution kernel size. Then, group convolution was performed using the 2D convolution of size *k* to index the position information on each channel dimension and fuse it, where *groups* = *C_i_*, recorded the output result as ${\hat{Z}}_{i}$ . Thus, the number of parameters of the PI can be calculated as:


(9) $$Params_{PI}=k^{2}C_{i}$$

the amount of computation can be calculated as:


(10) $$FLOPs_{PI}=k^{2}C_{i}WH$$

In addition, the PI was added after each Mix Transformer to index the absolute position information of the pixel points after establishing the global dependency.

Therefore, the PI indexed the absolute position information of the target dynamically. Such structure did not affect the translational invariance of the model and allowed the encoder to preserve the absolute position information while performing multi-scale downsampling. This helped to recover the position information accurately in the decoder and improve the segmentation performance of the model.

#### 2.3.3 Local optimization module

In the task of apple leaf disease segmentation, the proportion of disease pixels to the full image pixels was small, making the extraction of small disease features more difficult. In addition, the jagged features of the leaf edges were difficult to extract in complex environments, which can reduce the accuracy of leaf segmentation. The Mix Transformer we proposed in 2.3.1 was capable of global modeling, yet its ability to extract edge features was relatively weak and may miss small spots. Therefore, in this subsection, the LOM was designed to optimize the segmentation of leaf edges and extracted more tiny spots through using CNN’s ability to extract local features. The structure of LOM was presented in **Figure 3**.

As shown in **Figure 3**, the designed LOM first used a 1×1 convolution to up-dimension, then letted a 3×3 depthwise separable convolution perform local feature extraction in higher dimensions, and finally used a 1×1 convolution to adjust back to the initial dimension. By stacking multiple convolutions, the LOM can gradually capture different levels of texture features of the image and combine them to form a higher-level feature representation. Therefore, the LOM was added to the end of the Mix block to enhance the ability of the model to extract local features. Specifically, the ${\hat{Z}}_{i}$ output by the PI was used as input, the LOM can be expressed as:


(11) $${\hat{Z}}_{i}^{'}=Conv_{1\times 1}\left(DWConv_{3\times 3}\left(Conv_{1\times 1}\left({\hat{Z}}_{i}\right)\right)\right)$$

By optimizing the local features of the feature layer output from Mix Transformer, LOM can effectively enhance the model’s ability to extract detailed features, thus optimizing the segmentation effect of leaf edges and extracting more tiny spots.

## 3 Results

In this section, the experimental environment, hyper-parameter settings and dataset partitioning were presented in subsection 3.1. Then, the rationale and computational formulas for the evaluation metrics used in this study were presented in subsection 3.2. Thereafter, subsection 3.3 conducted ablation experiments to explore the effects of different sizes of MixSeg on segmentation performance. Next, subsection 3.4 compared the segmentation accuracy of different models on two outdoor apple disease leaf image datasets. Then, the segmentation results of different models in real scenes were visualized in subsection 3.5. Subsequently, the effective region of focus was analyzed in subsection 3.6.

### 3.1 Experiment setup

The hardware configuration of the experimental environment for this study was as follows: Intel(R) Core(TM) i5-12400F, 16 G memory, NVIDIA GeForce RTX3060ti, 64-bit Windows operating system. The model was built by the Pytorch framework; the version of PyTorch was 1.10.0. After several trials, the hyperparameters were set as follows: optimizer was Adamw, momentum was 0.9, weight decay was 1e-2, batch size was 8, the initial learning rate was 1e-4, the minimum learning rate was 1e-7, learning rate decay strategy was cos, drop path rate was 0.1, and epoch was 300.

We divided the two apple leaf datasets into a training set and a test set according to the 8:2 ratio. In addition, each dataset will be divided into a training set and a validation set according to 9:1 for cross-validation in the training phase.

### 3.2 Evaluation indicators

The following three evaluation metrics were selected to measure the segmentation effectiveness of the model: intersection over union (IoU), mean pixel accuracy (mPA) and pixel accuracy (PA).

In the segmentation task, IoU represents the ratio of the intersection and union among the predicted results of a category and the actual values of that category. mPA was the cumulative average of the proportion of pixels correctly classified in each category. PA represented the proportion of all correctly predicted pixels to all pixels. IoU, mPA and PA were calculated as follows:


(12) $$IoU=\frac{p_{ii}}{\sum_{j=0}^{k}p_{ij}+\sum_{j=0}^{k}p_{ji}-p_{ii}}$$

(13) $$mPA=\frac{1}{k+1}\sum_{i=0}^{k}\frac{p_{ii}}{\sum_{j=0}^{k}p_{ij}}$$

(14) $$PA=\frac{\sum_{i=0}^{k}p_{ii}}{\sum_{i=0}^{k}\sum_{j=0}^{k}p_{ij}}$$

where *k* +1 was the number of categories plus background, *p_ii _* was the number of correctly predicted pixels, *p_ij _* denoted the number of pixels belonging to category *i* but predicted as category *j*, and *p_ji _* denoted the number of pixels belonging to category *j* but predicted as category *i*.

### 3.3 Ablation studies

In this subsection, ablation experiments were performed on the model structure of MixSeg. The following tests were performed in the same experimental environment and utilized identical training parameters. In addition, total parameters, floating point operations (FLOPs), inference time, frames per second (FPS), IoU, mPA, and PA were used to evaluate the segmentation performance of the different models.

#### 3.3.1 Performance comparison between different versions of MixSeg

We designed four versions of MixSeg with different sizes by varying the output dimension of the model and the stacking number of Mix Transformer, which was named MixSeg-T, MixSeg-S, MixSeg-M, and MixSeg-L according to the complexity of the model. The results were used to analyze the effects of different output dimensions of each Mix stage and the number of Mix Transformer stacks on the model’s segmentation efficiency and segmentation accuracy. **Tables 1** and **2** demonstrated the segmentation performance comparison results of each version of the model.

**Table 1:** Performance metrics results for different versions of MixSeg.

Version	Output dimension	Mix Transformer stacking number	Total parameters/M	FLOPs/G	Inference time/ms	FPS
MixSeg-T	[32,64,160,256]	[2,2,2,2]	**1.90**	**4.17**	**8.70**	**114.82**
MixSeg-S	[32,64,160,256]	[4,4,4,4]	2.97	5.33	12.67	78.96
MixSeg-M	[64,128,256,512]	[2,2,2,2]	6.44	14.38	18.93	52.80
MixSeg-L	[64,128,256,512]	[4,4,4,4]	10.03	17.85	27.00	37.03

The bold font denotes which MixSeg version has the best performance on a particular evaluation metric.

**Table 2:** The results of segmentation accuracy for different versions of MixSeg.

	Apple alternaria blotch				Apple grey spot			
Version	IoU/%		mPA	PA	IoU/%		mPA	PA
	Leaf	Disease			Leaf	Disease		
MixSeg-T	98.22	87.40	97.21	99.45	98.09	86.20	97.26	99.46
MixSeg-S	98.39	88.55	97.54	99.50	98.32	87.98	97.62	99.53
MixSeg-M	98.41	88.63	97.58	99.51	98.40	88.12	97.67	99.55
MixSeg-L	**98.53**	**89.55**	**97.87**	**99.55**	**98.49**	**88.87**	**97.93**	**99.58**

The bold font denotes which MixSeg version has the best performance on a particular evaluation metric.

According to **Table 1**, increased the channel dimension of the Mix stage and the number of stacks of the Mix Transformer made the network more complex, reduced the efficiency of the network, and therefore it will lead to longer inference time and less FPS. Since the Mix Transformer we design was lightweight, increased the number of stacks of the Mix Transformer had less impact on the network complexity than increasing the channel dimension of the Mix stage. From **Table 3**, increased the network size can improve the segmentation accuracy on the test set of apple disease leaf images. In addition, the proposed lightweight models MixSeg-T and MixSeg-S were fast and efficient by the combined comparison of **Tables 1** and **2**, and they took into account the lightweight and maintain competitive performance in segmentation accuracy.

**Table 3:** The results of segmentation accuracy for different test models.

Test No.	Model	Apple alternaria blotch				Apple grey spot			
		IoU/%		mPA	PA	IoU/%		mPA	PA
		Leaf	Disease			Leaf	Disease		
1	Baseline	88.48	76.71	93.79	96.07	87.53	73.26	93.65	96.05
2	Baseline+PI	90.62	78.31	94.28	96.88	89.98	75.86	94.19	96.92
3	Baseline+LOM	97.48	84.42	96.48	99.21	97.36	82.22	96.18	99.25
4	Baseline+PI+LOM (Proposed method)	**98.22**	**87.40**	**97.21**	**99.45**	**98.09**	**86.20**	**97.26**	**99.46**

The bold font indicates which model performs best on a particular evaluation metric.

Thus, researchers can deploy MixSeg in different sizes for mobile devices in real leaf disease diagnosis scenarios depending on the specific situation. Generally, a larger MixSeg model will have higher segmentation accuracy but requires more computational resources and inference time. Therefore, researchers can choose a smaller MixSeg model for resource-constrained application scenarios to obtain faster inference speed and less computational resource consumption. In contrast, they can choose a larger MixSeg model for high-precision segmentation scenarios to obtain higher segmentation accuracy.

#### 3.3.2 Performance verification of PI and LOM

We designed four sets of experiments to verify the performance of the proposed PI and LOM. Specifically, Test 1 used MixSeg-T as the base framework and removes the PI and LOM, which were set as the baseline. Test 2 and Test 3 were both based on Test 1, which introduced the PI and LOM separately. In addition, Test 4, which was the method proposed in this paper, introduces both the PI and the LOM on top of Test 1. The evaluation results of the different test models were shown in **Tables 3**, **4** were shown.

**Table 4:** Performance metrics results for different test models.

Test No.	Model	Inference time/ms	FPS	Total parameters/M	FLOPs/G
1	Baseline	**7.00**	**142.69**	**1.70**	**3.99**
2	Baseline+PI	7.37	135.68	1.71	4.01
3	Baseline+LOM	8.48	120.32	1.89	4.15
4	Baseline+PI+LOM (Proposed method)	8.70	114.82	1.90	4.17

The bold font indicates which model performs best on a particular evaluation metric.

As shown in **Table 3**, by compared Test 1 and Test 2, the model with the introduction of PI outperformed the baseline model in segmentation accuracy. On apple alternaria blotch and apple grey spot test sets, the IoU for leaf segmentation was improved by 2.14% and 2.45%, respectively. The IoU for lesion segmentation was improved by 1.6% and 2.6%, respectively. Compared to Test 1 and Test 3, the segmentation accuracy of the model was improved more significantly compared to the baseline model after the introduction of LOM. The IoU for leaf segmentation was improved by 9.00% and 9.38%, respectively. The IoU for lesion segmentation was improved by 7.71% and 8.96%, respectively. The results indicate that PI helps to accurately recover the position information of the target leaf, lesion in the decoder due to its ability to dynamically index the absolute position information of the target, which in turn improves the segmentation effect of the model. And LOM can optimize the segmentation effect of leaf edges and extract more tiny spots, so it can also improve the segmentation effect of the model. In comparison with Test 4 and other tests, the combination of both PI and LOM, introduced on the baseline model, gave the best performance in leaf and spot segmentation. The results demonstrate that the proposed method is designed to maximize the segmentation accuracy of the model as the LOM is able to accurately and efficiently optimize the edge features of the target leaves and diseases after the PI indexes to the absolute position information of the target leaves and spots. In addition, observed **Table 4**, the effects of PI and LOM on model inference time, total parameters and FLOPs were relatively minor. However, compared Test 1 and Test 4, the proposed method decreased 27.87 in FPS compared to the baseline model due to the increase in network complexity. Due to the complexity of the orchard environment, sacrifice of some FPS to ensure accurate segmentation of apple leaves and diseases was necessary. In summary, the proposed method had good segmentation performance in the task of segmenting apple diseased leaf images.

### 3.4 Comparsion of different models

In this subsection, to demonstrate the advantages of the MixSeg model, MixSeg was compared with popular deep learning semantic segmentation models, including CNN-based and Transformer-based single structure models. These models were PSPNet (Zhao et al., 2017), HRNetV2 (Sun et al., 2019), U-Net (Ronneberger et al., 2015), DeepLabv3+ (Chen et al., 2018), and SegFormer (Xie et al., 2021b). PSPNet used a pyramid pooling module to efficiently exploit contextual information. HRNetV2 provided rich semantic information by simultaneously extracting features at branches of different resolutions and then fusing features from these branches to maintain high resolution while providing rich semantic information. U-Net used a symmetric encoding-decoding structure to achieve multi-scale information fusion by jump linking. DeepLabv3+ used atrous spatial pyramid pooling (ASPP) to efficiently enhance the receptive field and mitigate the information loss problem caused by pooling. SegFormer was a Transformer semantic segmentation-based model, which further improves the segmentation efficiency of the model by reducing the number of parameters of self-attention and decoder. To be fair, all models were placed in the same experimental environment as MixSeg, and both were trained on two outdoor apple disease leaf image datasets. The segmentation accuracy of different models was shown in **Table 5**.

**Table 5:** The results of segmentation accuracy of different models on two diseased leaf test sets.

Model	Apple alternaria blotch				Apple grey spot			
	IoU/%		mPA	PA	IoU/%		mPA	PA
	Leaf	Disease			Leaf	Disease		
PSPNet	95.47	71.80	93.50	98.56	94.69	64.84	91.91	98.46
HRNetV2	95.12	79.23	94.06	98.45	95.75	76.85	93.92	98.77
SegFormer	96.80	82.22	95.89	99.00	96.60	78.94	94.93	99.03
U-Net	95.49	83.72	95.71	98.57	95.46	82.23	95.99	98.70
DeepLabv3+	96.85	82.55	96.74	99.01	96.47	79.41	96.88	98.98
MixSeg-T	**98.22**	**87.40**	**97.21**	**99.45**	**98.09**	**86.20**	**97.26**	**99.46**

The bold font indicates which model performs best on a particular evaluation metric.

As illustrated in **Table 5**, MixSeg-T performed best on both outdoor apple diseased leaf image datasets. Taking the apple alternaria blotch leaf dataset as an example, the same results were shown on another dataset. MixSeg-T was much better than PSPNet in segmentation accuracy. Compared with PSPNet, MixSeg-T achieved 2.75% higher IoU for leaf segmentation and 15.6% higher IoU for lesion segmentation. HRNetV2 had less accuracy than MixSeg-T in both leaf and disease spot segmentation, with the IoU of leaf segmentation being 3.1% lower than that of MixSeg-T and the IoU of disease spot segmentation being 8.17% lower than that of MixSeg-T. Compared to SegFormer, MixSeg-T achieved 1.42% higher IoU for leaf segmentation and 5.18% higher IoU for lesion segmentation. In addition, DeepLabv3+ and U-Net were closer and better than other methods in segmentation accuracy, but both were inferior to MixSeg-T. DeepLabV3+ was 1.37% and 4.85% lower than the proposed method in the IoU of leaf and spot segmentation, respectively. At the same time, U-Net was 2.73% and 3.68% lower in the IoU of leaf and spot segmentation, respectively.

This indicates that the combined CNN, Tranformer and MLP models outperform the single structure model in terms of segmentation accuracy for apple leaves and diseases in outdoor environments. Therefore, the more accurate segmentation capability of MixSeg can help growers better analyze the disease status of their crops, detect pests and diseases in time and take effective preventive measures. It can also help researchers gain a deeper understanding of the characteristics and development patterns of crop diseases and improve the accuracy and efficiency of disease diagnosis.

**Table 6** recorded the inference time, FPS, total parameters and FLOPs for all models. According to **Table 6**, MixSeg-T exhibited a more lightweight performance. MixSeg-T was 30.97ms faster than PSPNet in inference time, and the FPS was 89.62 higher. Moreover, the total parameters and FLOPs of MixSeg-T were only 3.9% and 6.8% of PSPNet. The inference time of HRNetV2 was 20.87ms slower than MixSeg-T, and the FPS was 81.01 less. Compared with the lightweight SegFormer, the inference time of MixSeg-T was 1.84ms faster, the FPS was 20.02 higher, and the total parameters and FLOPs are lower than SegFormer. In contrast to MixSeg-T, the inference time of U-Net was 36.06ms slower than MixSeg-T, and the FPS was 92.49 lower. Moreover, the total parameters and FLOPs of MixSeg-T were only 7.6% and 1.8% of U-Net. In addition, the inference speed and FPS of DeepLabv3+ were close to those of MixSeg-T, but MixSeg-T had fewer total parameters and FLOPs.

**Table 6:** The results of performance metrics for different models.

Model	Inference time/ms	FPS	Total parameters/M	FLOPs/G
PSPNet	39.67	25.20	49.07	61.63
HRNetV2	29.57	33.81	9.64	18.66
SegFormer	10.54	94.80	3.71	6.77
U-Net	44.76	22.33	24.89	225.85
DeepLabv3+	**7.96**	**125.52**	5.81	26.44
MixSeg-T	8.70	114.82	**1.90**	**4.17**

The bold font indicates which model performs best on a particular evaluation metric.

In practical scenarios, apple trees in outdoor environments can be widely dispersed, and the diagnosis of leaf diseases needs to be conducted at various locations. This necessitates models with smaller scale and lower computational requirements to be optimally deployed on mobile devices, enhancing the flexibility and efficiency of the diagnosis process. Experimental findings demonstrate that MixSeg outperforms mainstream segmentation models in terms of lightweight characteristics, rendering it more suitable for real-world applications. It can be swiftly deployed on mobile devices such as smartphones, drones, and application robots, enabling efficient diagnosis of leaf diseases. Consequently, this facilitates the timely implementation of effective preventive measures.

### 3.5 Visualization of apple diseased leaf segmentation results

To validate the segmentation performance of MixSeg in complex environments, all models were predicted with two sets of outdoor apple disease leaf test images. We adjusted the transparency of the predicted result image to 50% and then fused it with the original image to compare the segmentation effect of each method more clearly.

**Figure 6:**
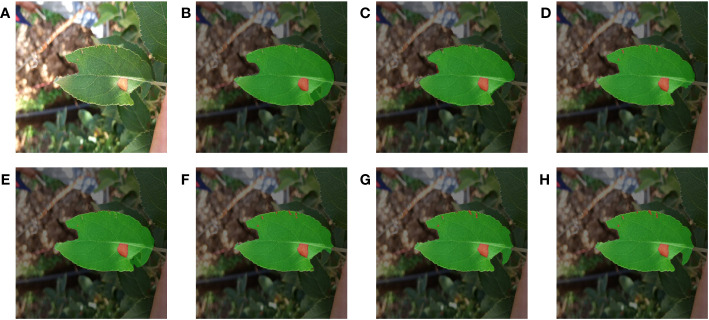
Fusion segmentation results of different models for apple alternaria blotch leaf with breakage. **(A)** Image. **(B)** PSPNet. **(C)** HRNetV2. **(D)** SegFormer. **(E)** DeepLabv3+. **(F)** U-Net. **(G)** MixSeg-T. **(H)** MixSeg-L.

**Figure 7:**
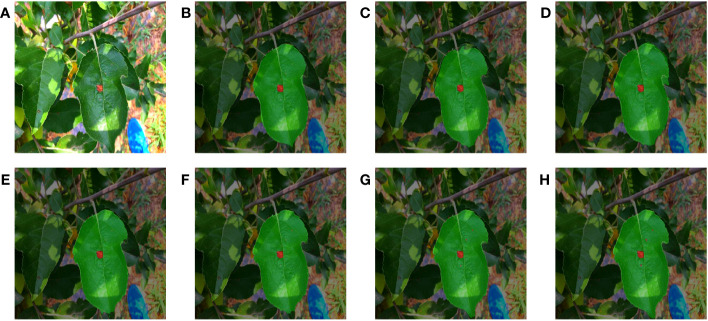
The results of fusion segmentation of different models on overlapping apple alternaria blotch leaves. **(A)** Image. **(B)** PSPNet. **(C)** HRNetV2. **(D)** SegFormer. **(E)** DeepLabv3+. **(F)** U-Net. **(G)** MixSeg-T. **(H)** MixSeg-L.

**Figures 6** and **7** demonstrated the segmentation results of each model under the test set of apple alternaria blotch leaf images. Compared **Figure 6**, PSPNet was less effective in segmenting outdoor leaves and was easily disturbed by other leaves. Moreover, PSPNet missed many small disease spots. This poor performance can also be seen in **Figure 7**. HRNetV2 did not segment both leaves and disease spots well. The leaf area extracted by HRNetV2 is incomplete, and small spots were missed. SegFormer was less effective in outdoor leaf image segmentation, and there was a loss of tiny spots. DeepLabv3+ was not accurate enough in extracting leaf regions and misses small spots. Although the segmentation of U-Net was better and superior to the previously mentioned methods, it also suffered from the interference of overlapping leaves and misses some tiny spots. We can see that both MixSeg-T and MixSeg-L have better segmentation results than U-Net. They can extract the target leaves clearly from the complex environment and the tiny spots missed by other methods.

**Figure 8:**
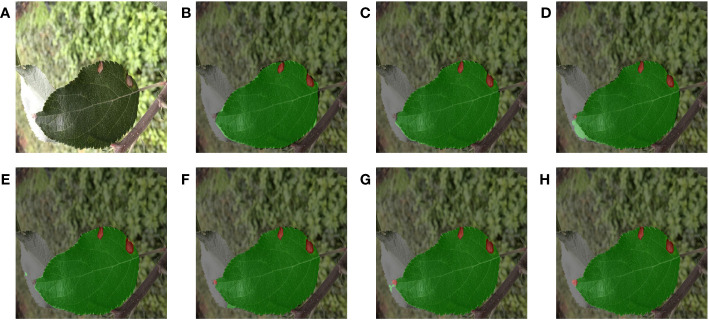
The results of different models for fusion segmentation of apple gray spot leaves with lesions present at the leaf tip. **(B)** PSPNet. **(C)** HRNetV2. **(D)** SegFormer. **(E)** DeepLabv3+. **(F)** U-Net. **(G)** MixSeg-T. **(H)** MixSeg-L.

**Figure 9:**
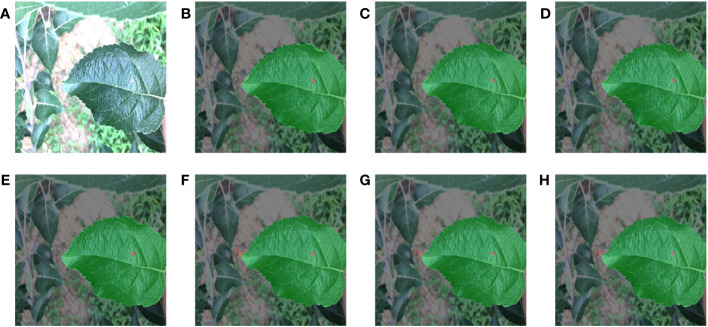
The results of fusion segmentation of apple gray spot leaves by different models in overilluminated environments. **(B)** PSPNet. **(C)** HRNetV2. **(D)** SegFormer. **(E)** DeepLabv3+. **(F)** U-Net. **(G)** MixSeg-T. **(H)** MixSeg-L.

**Figures 8** and **9** demonstrated the segmentation results of each model under the test set of apple grey spot diseased leaf images. Compared **Figures 8**, **9**, PSPNet can usually segment the whole target leaf, but it was vulnerable to other environmental factors. Therefore the target leaf area extracted by PSPNet was not complete enough to segment the serrated shape of the leaf edge. Moreover, the color of the disease spots at the leaf tip was similar to some elements in the background, and PSPNet cannot extract these spots. The leaf areas extracted by HRNetV2 were somewhat more complete than those of PSPNet. However, HRNetV2 also cannot segment the serrated shape of the leaves. Moreover, HRNetV2 also missed the disease spots at the leaf tips. SegFormer had poor segmentation performance on leaves, which were disturbed by other leaves. In addition, SegFormer could not accurately extract the diseased areas at the leaf tips. DeepLabv3+ was also less effective in segmenting the spots at the leaf tips. The segmentation of U-Net was better and superior to the above methods, but not as good as MixSeg-T and MixSeg-L. Although U-Net extracts the spots at the leaf tips, the area of the disease spots was not accurate enough. In contrast, MixSeg-T and MixSeg-L can extract the area of leaf tip spots more accurately. In addition, MixSeg-T and MixSeg-L can better segment the serrations on the leaf edges.

**Figure 10:**
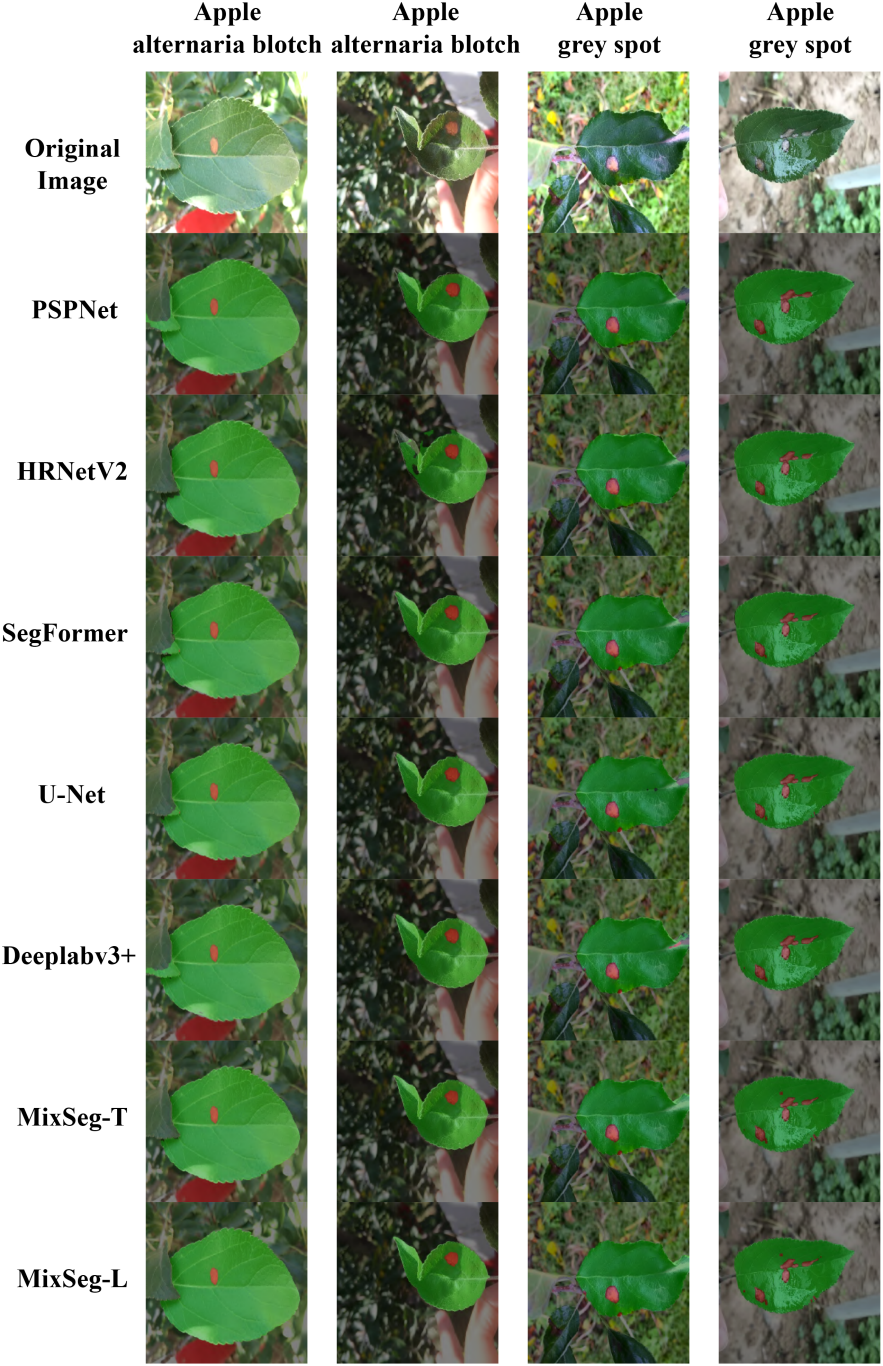
Fusion segmentation results of different models for outdoor apple alternaria blotch and grey spot leaf image datasets.

To more comprehensively compare the differences in segmentation performance between the different models, **Figure 10** compared the segmentation results. From **Figure 10**, the comparison with MixSeg, other models had poor segmentation results due to the difference in feature extraction structure. For example, the pyramid pooling and spatial pyramid structure of PSPNet and DeepLabv3+, although expanding the sensory field, tended to miss small spots in the downsampling. SegFormer, although used self-attention for global modeling, cannot encode the absolute position it cannot accurately recover the position information of leaves and spots in the upsampling. This led to poor segmentation results. Through a comprehensive comparison, MixSeg outperformed other methods in terms of segmentation effect.

### 3.6 Effective focus on regional analysis

**Figure 11:**
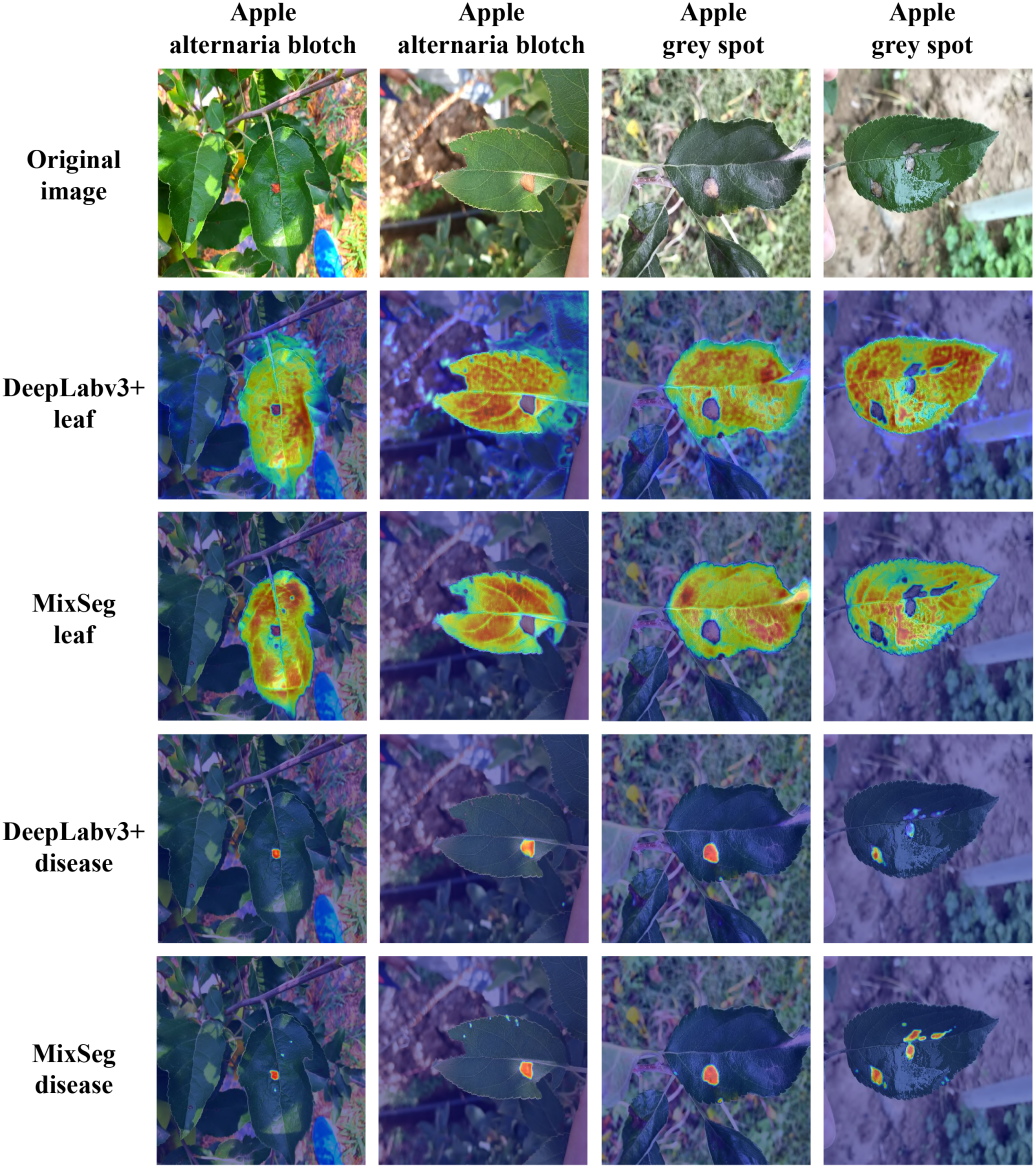
The results of DeepLabv3+ and MixSeg for effective focus areas on outdoor leaves and diseases. Areas with brighter colors or higher intensity in the heat map are considered more important for network decision-making, while areas with darker colors or lower intensity are relatively less important. These focus areas play an important role in the final prediction of the network.

To further demonstrated the effectiveness of the MixSeg architecture, we outputted the heat map of effective attention regions for DeepLabv3+ and MixSeg classification heads used the Grad-CAM technique separately and compared them. DeepLabv3+ was selected because it can be found through various experiments that DeepLabv3+ had the best overall performance except for MixSeg. Grad-CAM explained the parts of the network that are of concern in the decision-making process, and these regions of concern played an essential role in the final prediction. Regions with brighter colors or higher intensities in the heat map were considered more important for the network’s decision-making. In contrast, regions with darker or lower intensities were relatively less critical. Based on this, we can better measure the segmentation performance of the network. **Figure 11** showed the comparison results of DeepLabv3+ and MixSeg effective attention regions.

Observed the second and third rows of **Figure 11**, the effective area of focus of MixSeg can accurately focus on the target leaves. However, DeepLabv3+ was not focused enough on the target leaf because it was vulnerable to environmental distracting factors. In addition, through comparing with DeepLabv3+, MixSeg can notice the serrated shape on the leaf edge so that it can obtain better leaf segmentation results. Compared with the fourth and fifth rows of **Figure 11**, the color of the spots was similar to the color of some elements in the background, and DeepLabv3+ was less resistant to interference and therefore misses the attention to some spots. In contrast, MixSeg can focus on the spots missed by DeepLabv3+. Moreover, MixSeg was able to give more substantial attention to the spots. Therefore, MixSeg had a better segmentation effect on the spots in the final prediction. In summary, MixSeg was more resistant to interference and had a more accurate region of attention, which made MixSeg have better segmentation performance.

## 4 Discussion

The MixSeg model proposed in this paper introduced a hybrid structural network model based on CNN, Transformer and MLP for apple leaf and disease segmentation. By comparing with the latest segmentation models, it can be found that MixSeg was the most lightweight with the best segmentation accuracy and segmentation effect. In addition, the segmentation performance of MixSeg with different sizes was verified by ablation experiments. The ability of MixSeg to accurately extract leaves and diseases in complex environments was further demonstrated by analyzing and comparing the effective regions of focus of MixSeg and DeepLabv3+.

In the current work, the feasibility of MixSeg applied to segment apple leaf diseases in real scenarios was deeply experimented and discussed. MixSeg was designed to be lightweight for the requirements of deployment on mobile devices in real scenarios. In addition, MixSeg was resistant to interference and had strong feature representation capability and flexibility to capture and learn the features of target leaves and diseases in complex environments. Although the performance of MixSeg had only been validated on the task of apple leaf spot segmentation in complex environments in this study, the core principles and technical framework were somewhat generalizable and therefore very likely to be applied to other crop leaves. In the subsequent work, we will further add different varieties of crop disease leaf datasets and continue to train MixSeg in order to improve its generalization ability in practical applications. However, there may be some limitations when applying MixSeg to other crop leaf categories. For instance, different kinds of leaves may have a varying morphology, texture and disease characteristics, which may limit the performance of MixSeg in some cases. Therefore, in future research, when extending MixSeg to other crop leaves, further research and understanding of the characteristics of the target crop leaves and diseases are needed to optimize and adjust accordingly to ensure the accuracy and robustness of the method.

## 5 Conclusion

This work presented a mixed-structure semantic segmentation method, MixSeg, for fast and accurate segmentation of apple leaves and diseases in complex environments. In this model, Mix Transformer was designed to inject multi-scale local biases into the model at a much smaller computational cost and establishes global dependencies between pairs of pixels. The PI was proposed, which was independent of the input length and thus allowed the model to perform multi-scale modeling while extracting learnable absolute position information. LOM was proposed to optimize the effect of the model on local feature extraction. In addition, the advantages of the mix structure model were demonstrated by comparing various experiments of MixSeg with mainstream single-structure semantic segmentation models. The experimental results showed that MixSeg was the most effective in segmenting apple diseased leaf images in complex environments, which made this study a key attempt to advance the research on smart agriculture. However, only apple diseased leaves were selected as experimental objects in the study, and the generalization ability of the model needed to be further improved. In future research, we will increase the variety of datasets used for training and further optimize the model according to the characteristics of different crop diseased leaves and the complex environments in which they grow, so that the mix structure model can be more widely applied to the field of smart agriculture.

## Data availability statement

The raw data supporting the conclusions of this article will be made available by the authors, without undue reservation.

## Author contributions

BL and JL contributed to conception and design of the study. JL, XX, and YJ contributed to the preparation of the equipment and the acquisition of the data. JL wrote the code and tested the method. JL and XX carried out the experiments and verified the methods. JL wrote the manuscript. BL were responsible for checking and revising the manuscript. All authors contributed to the article and approved the submitted version.
